# Multifunctional Roles of miR-34a in Cancer: A Review with the Emphasis on Head and Neck Squamous Cell Carcinoma and Thyroid Cancer with Clinical Implications

**DOI:** 10.3390/diagnostics10080563

**Published:** 2020-08-05

**Authors:** David Kalfert, Marie Ludvikova, Martin Pesta, Jaroslav Ludvik, Lucie Dostalova, Ivana Kholová

**Affiliations:** 1 Department of Otorhinolaryngology and Head and Neck Surgery, University Hospital Motol, 1st Faculty of Medicine, Charles University, 15006 Prague, Czech Republic; david.kalfert@fnmotol.cz (D.K.); lucie.dostalova2@fnmotol.cz (L.D.); 2 Department of Biology, Faculty of Medicine in Pilsen, Charles University, 32300 Pilsen, Czech Republic; martin.pesta@lfp.cuni.cz; 3 Department of Imaging Methods, University Hospital Pilsen, Faculty of Medicine in Pilsen, Charles University, 32300 Pilsen, Czech Republic; ludvikj@fnplzen.cz; 4 Faculty of Medicine and Health Technology, Tampere University, 33520 Tampere, Finland; ivana.kholova@tuni.fi; 5 Pathology, Fimlab Laboratories, 33520 Tampere, Finland

**Keywords:** miR-34a, head and neck squamous cell carcinoma, thyroid cancer

## Abstract

MiR-34a belongs to the class of small non-coding regulatory RNAs and functions as a tumor suppressor. Under physiological conditions, miR-34a has an inhibitory effect on all processes related to cell proliferation by targeting many proto-oncogenes and silencing them on the post-transcriptional level. However, deregulation of miR-34a was shown to play important roles in tumorigenesis and processes associated with cancer progression, such as tumor-associated epithelial-mesenchymal transition, invasion, and metastasis. Moreover, further understanding of miR-34a molecular mechanisms in cancer are indispensable for the development of effective diagnosis and treatments. In this review, we summarized the current knowledge on miR-34a functions in human disease with an emphasis on its regulation and dysregulation, its role in human cancer, specifically head and neck squamous carcinoma and thyroid cancer, and emerging role as a disease diagnostic and prognostic biomarker and the novel therapeutic target in oncology.

## 1. Introduction

MicroRNAs (miRNAs) are short non-coding single-stranded RNA with 18–25 nucleotides in length. The expression of 2654 mature human miRNAs has been reported in the latest available miRNA database (miRBase release 22; http://www.mirbase.org/) [1]. They have important regulatory functions such as the control of the expression of the structural genes. Thus, the expression of more than 30% of human structural genes is controlled by miRNAs at the post-transcriptional level by binding to a 3′ untranslated region (3′ UTR) of target messenger RNAs (mRNA). In addition, miRNAs regulate gene expression via translational repression or mRNA destabilization. In rare instances, it can also cause translational activation [2]. It is now established that the secondary structure of the 5′ untranslated region (5′ UTR) of mRNA is important for microRNA-mediated gene regulation in humans [3]. In this way, miRNAs can modulate the expression of target genes and, consequently, many important biological processes, such as cell proliferation, differentiation, apoptosis, autophagy, immune response, etc. Some miRNAs are tissue-type and cell-type specific [4].

Recently, miR-34a was an oncological research hot spot. MiR-34a is one of the three members of the miR-34 family along with miR-34b and miR-34c [5]. MiR-34a attracted the attention of researchers in 2007, when the miR-34 family was originally cloned and characterized as a p53 target gene [6,7]. MiR-34a is ubiquitously expressed whereas miR-34b/c is mainly expressed in lung tissue [8].

In this review, we summarized the current knowledge on miR-34a functions in human disease with an emphasis on its regulation and dysregulation, its role in human cancers, specifically head and neck squamous carcinoma (HNSCC) and thyroid cancer, and an emerging role as a disease biomarker and the novel therapeutic target in oncology.

## 2. Overview of miR-34a Biogenesis and Regulations of miR-34a Expression

### 2.1. MiR-34a Biogenesis

**Figure 1:**
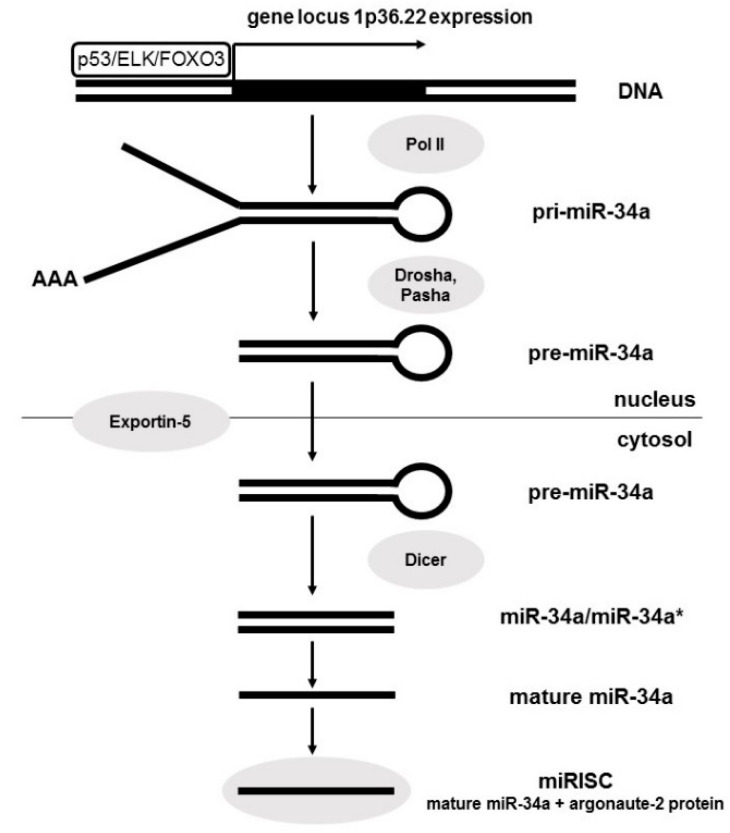
MiR-34a biogenesis.

The miR-34a gene locus is at chromosome 1p36.22. This gene does not encode any other non-coding RNAs or proteins. MiR-34b and miR-34c have a common gene on the chromosome 11q23.1 and are produced by a common primary transcript. The expression of miR-34a is ubiquitous while miR-34b and miR-34c are predominantly produced in the lung [9,10]. The biogenesis of miRNA begins in the nucleus and matures in the cytoplasm. The generation of mature miR-34a is similar to the other miRNAs multistage process, which include the following steps (Figure 1). First, the miR-34a encoding gene is transcribed by RNA-polymerase II to produce the primary transcript (pri-miR-34a) in the nucleus. The crucial transcription factors inducing the expression of this gene are p53, ELK, and FOXO3. The next step is the first cleavage of pri-miR-34a by the RNAse III enzyme Drosha (which is also stimulated by p53) and, subsequently, Pasha into a special stem-loop-structure called a precursor molecule miR-34a (pre-miR-34a). This molecule is transported to the cytoplasm by Exportin 5. The last step is second cleavage by RNase III enzyme Dicer to create a short RNA duplex consisting of the mature miRNA and its anti-sense strand (miRNA*). Lastly, mature miRNA-34a is deliberated and loaded together with argonaute proteins into the RNA-induced silencing complex (RISC). This complex mediates the interaction between the miRNA and the target mRNA to mediate its direct silence [10,11].

### 2.2. p53 Dependent miR-34a Regulation

The canonical induction of miR-34a by protein p53 was documented by many researchers [12,13,14,15,16,17]. The tumor suppressor protein p53 functions as a transcription factor that regulates the expression of several miRNAs including miR-34 and family. MiR-34 gene promoters contain p53 binding sites. Notably, miR-34a is the important component of the p53 complex regulatory network [12,13,14,15]. MiR-34a is implicated in the control of many cancer-related processes and it has been validated as the important mediator of the tumor suppressive function of p53 after DNA damage by suppressing its target mRNAs, such as Bcl-2, SNAIL, etc. [16]. While p53 induces the transcription of miR-34a, this one can regulate the expression and activity of the p53 protein in a positive and negative manner. This positive feedback loop between p53 and miR-34a is mediated by post-transcriptional suppression of proteins MDM4 (and its human homolog HDM4) and/or SIRT1 expression to enhance p53 transcriptional activity and stability. Both these proteins under physiological conditions inhibit the transcriptional activity of TP53 and they are targets of miR-34a. On the contrary, the negative effect of miR-34a on gene TP53 and some of p53 pathway-associated genes may consist in direct targeting their mRNAs [17,18,19]. Navarro and Lieberman documented the enhanced p53 transcription activity in cultured cells due to miR-34a overexpression and explained a complex functional relationship between miR-34a and p53. In comparison with miR-34a, there is only a weak effect of miR-34b/c overexpression on p53 function [19].

Protein p53 offers protection against an uncontrolled proliferation of cells with damaged DNA, initiation of DNA repair, and apoptosis. Some of its functions are mediated through miR-34a. However, p53 exerts its function even in the absence of miR-34a. In such a case, all functions are usually mediated through the other miRNAs, such as the miR-449 family, that has the same seed site with miR-34a [19]. The interplay and cooperation between miR-34a and the protein p53 seem to be very complex. On one hand, the miR-34a is transcriptionally activated by p53. The genomic mutation at the p53-binding site in the miR-34a gene may cause the loss of miR-34a expression [20]. Contrarily, the gene TP53 is a direct target of miR-34a even though the molecular mechanism of the influence of miR-34a on p53 in humans has not been described.

**Figure 2:**
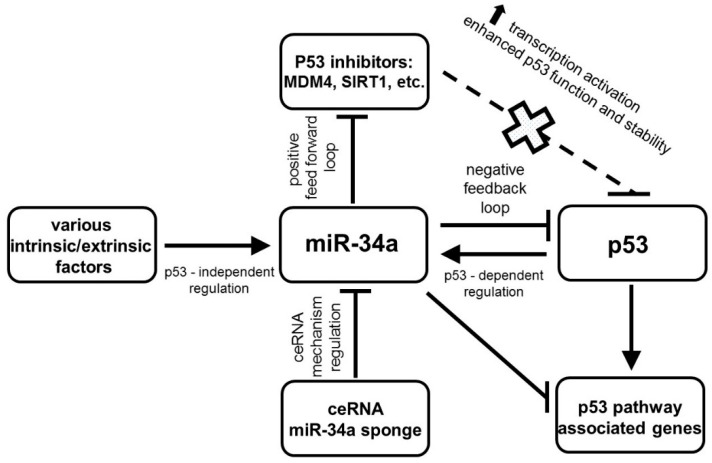
Regulatory mechanisms of miR-34a expression.

The tumor suppressor effects of miR-34a is mediated through both p53 dependent (canonical) and p53-independent (non-canonical) mechanisms [19] (Figure 2).

### 2.3. p53 Independent miR-34a Regulation

Besides a p53-dependent pathway of miR-34a regulation, miR-34a expression was found to be driven independently of p53 as well, especially in the case of disrupted p53 function [19,21]. The p53-independent mechanism is associated with the detection of miR-34a in tissues with gene TP53 disrupted by mutation or inactivated by viral proteins. In such cases, the basal level of miR-34a expression can be maintained by p53-independent mechanisms regardless of altered or blocked p53 function. The protein p53-independent manner of the miR-34a enhancement is implicated both in physiological conditions and in pathological processes.

The crucial initiation factors of p53-independent regulation of miR-34a are classified into intrinsic (cellular) and extrinsic (microenvironmental, extracellular) [21]. The former group is represented by mechanisms as epigenetic changes or altered cancer-related signaling with oncogene activation. The latter one is associated with hypoxia, inflammation, cellular plasticity/epithelial mesenchymal transition (EMT), or an immune response as well. In respect to epigenetic changes, miR-34a expression is induced by promoter hypomethylation since it was documented in the liver with tissue regeneration. Similarly, the upregulation of miR-34a expression may be caused in cancer [22]. Constitutive activation of the oncogene may result in oncogene-induced cellular senescence that is an irreversible growth arrest serving as a barrier against tumorigeneses. The effect of different oncogenes to induce senescence likely depends on the cell and tissue type. The underlying mechanisms have not been entirely elucidated [23]. MiR-34a is involved in this process and its upregulation by transcription factor ELK1 during B-RAF-induced senescence was described. In addition, ELK1 belongs to the members of ETS family that is able to activate the tumor suppressor p16INK4a promoter. Subsequently, miR-34a targets oncogene MYC to repress its proliferative function and, thus, trigger senescence [23].

### 2.4. ceRNA Network in miR-34a Regulation

A novel, recently described regulation of miRNA levels and, thereby, target gene expression was shown in the study of Ebert et al. in 2007 [24]. Subsequently, the principle of novel conception referred to as competing endogenous RNA (ceRNA) hypothesis was proposed by Salmena et al. in 2011. This theory suggests that RNAs can regulate each other by competing for a limited pool of miRNAs [25]. Simultaneously, a new class of non-coding RNAs was introduced. These RNAs compete with specific mRNA to provide specific binding sites called “microRNA response elements“ (MREs) to the corresponding miRNAs. Thus, the concentration of specific miRNA can be paradoxically temporally reduced in the specific cell, which results in an upregulated expression of the target mRNA. CeRNAs, acting as miRNA sponges, include transcripts such as circular RNA (circRNA), long non-coding RNA (lncRNA), some mRNA, and pseudogenes that can cross-talk each other and with coding RNAs through miRNAs. Some authors confirmed that the ceRNA network plays key regulatory roles in many biological processes such as cancer [26,27].

Among miRNAs involved in the ceRNA network, miR-34a occupies an important position, which was documented recently in several studies [28,29,30,31]. Some miR-34a targets have been recently shown to control the function of miR-34a through the ceRNA mechanism. CircRNA, lncRNA, and some mRNA as members of the ceRNA family might act as an miR-34a sponge to upregulate cancer progression. For instance, He et al. revealed circGFRA1 sponging of miR-34a to upregulate the progression of triple-negative breast carcinoma cell lines and tissue [28]. Similarly, lnc015192 and lncRNA FEZF1-AS1 act as miR-34a sponges to upregulate ADAM1 expression in breast cancer cells and Notch-1 in glioblastoma, respectively, and, thereby, promoting cancer cell migration [29,30]. C-mycRNA targeting miR-34a acts as a ceRNA and upregulates CD44 expression in urothelial carcinoma [31].

## 3. Overview of Functions of miR-34a

**Figure 3:**
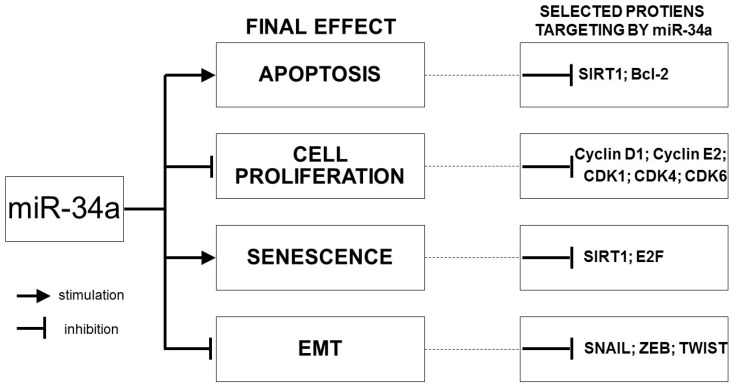
Normal regulatory role of miR-34a in cancer-relevant pathways and selected proteins targeting by miR-34a involved in these processes.

Generally, the principal function of miRNAs is post-transcriptional silencing of target genes. MiRNA binds to the 3′ untranslated region (3′-UTR) target mRNA in a complete or an incomplete complementary manner in the 5′ untranslated region to exert downregulation and/or degradation of it, and, thereby, inhibits target gene expression. The mechanism of mRNA silencing depends on the degree of complementarity with the target mRNA. Simultaneously, the 3′-UTR of most mRNA has more than one binding site for a different miRNA [2]. MiR-34a has more than 700 validated gene targets [32]. Most of these genes are essential for normal survival and development [4]. MiR-34a can control the expression of many target genes through their mRNAs and, thus, can be implicated in a plethora of important biological processes, particularly in cell proliferation, differentiation, apoptosis, and regulation of migration [7,21,33,34]. Some processes are stimulated (differentiation, apoptosis) while the other ones are inhibited (cell cycle, stemness, or EMT) [10]. MiR-34a, as a tumor suppressor, has a negative effect on all processes associated with cancer progression such as tumor-associated EMT, invasion, and metastasis [35] (Figure 3).

Apoptosis is the process in which regulation by miR-34a is involved through several proteins, among them the proteins B-cell lymphoma 2 (Bcl-2) and silent information regulator (SIRT1), which play an important role. Both these proteins have the anti-apoptotic function and serve as direct targets of miR-34a that represses their translation. SIRT1 inhibition, thus, results in an increase of p53 acetylation, stabilization, and, thus, in p53 transcriptional activation, which is followed by the gain of p53-dependent apoptosis in response to cell damage. Downregulation of bcl-2 expression by miR-34a mediates the initiation of apoptosis by the activation of pro-apoptotic proteins Bax and Bak [5,17,20,36].

Cell proliferation is influenced by miR-34a through many proteins by regulating the cell cycle, such as cyclins and cyclin dependent-kinases (cyclin D1, cyclin E2, CDK4, CDK6, CDK1, CDC25C phosphatases, etc.) that promote cells to proceed through the G1 phase into the S phase and through the G2 phase to the M phase, respectively. Silencing them using miR-34a inhibits the cell cycle through arrest in the G1 or G2 phase [20].

Cellular senescence is a stress-induced irreversible cell cycle arrest and other phenotypic alterations in normal cells. MiR-34a induces senescence via miR-34a-SIRT1-p53 and miR-34a-E2F-RB axes by downregulation of SIRT1 and E2F proteins. The SIRT1 protein under normal conditions mediates deacetylation of p53 and, thus, inhibits p53 mediated senescence and apoptosis [20].

Tumor-associated processes (epithelia-mesenchymal transition, cancer invasion, and metastasis) are crucial mechanisms underlying tumor progression. Epithelial to mesenchymal transition (EMT) refers to the loss of the epithelial phenotype of cells and the gain of the mesenchymal features at the invasive front of cancer and the expression of mesenchymal markers such as N-cadherin, vimentin, fibronectin, and matrix metalloproteinases. Tumor-associated EMT is the principal mechanism underlying cancer progression, invasiveness, and metastatic ability [37]. MiR-34a acting in cancer as a tumor suppressor negatively regulates EMT predominantly by inhibiting EMT-associated transcription factors (EMT-TFs) such as SNAIL, ZEB, and TWIST. Moreover, miR-34a controls EMT-signaling pathways, e.g., Wnt signaling, Notch signaling, and TGF-beta1/Smad signaling. The role of ceRNA in EMT by sponging miR-34a was revealed as well. In these ways, miR-34a can inhibit or stimulate cancer progression and invasion [35]. Contrarily, miR-34a expression is reciprocally controlled by EMT-regulatory molecules, which results in repression of miR-34a transcription [38].

Chronic inflammation may trigger cancer transformation and progression via several secreted molecules, particularly TNF-alfa and IL-6. Both these molecules are negatively regulated by miR-34a to exert its anti-inflammatory and antitumoral effect. MiR-34a was shown to be an important regulator of programmed cell death ligand 1 (PD-L1) and, consequently, regulator of the immune response in cancer. MiR-34a binds PD-L1 mRNA and blocks PD-L1 expression on the cell membrane. Lower expression of miR-34a in the tumor is usually associated with TP53 mutation and high PD-L1 expression, which develops a treatment option via immune checkpoint inhibition [39].

## 4. Aberrant Expression of miR-34a in Cancer

MiRNAs with their wide scale of functions are implicated in a number of physiological processes and in pathological conditions as well. The physiological miRNAs inhibitory function helps to regulate the normal gene expression in the cell. However, dysregulated miRNA expression underlies many diseases. For instance, inappropriate apoptosis, either decreased or increased, is the essential pathobiological mechanism in many human conditions, such as neurodegenerative diseases, ischemic damage, autoimmune diseases, and many types of neoplasms [7,20]. Due to dysregulation, miRNAs are classified into two types—oncogenic and tumor suppressor-according to their distinct predominant functions in cancers. Oncogenic miRNAs (OncomiRNAs) are upregulated in cancer cells and contribute to carcinogenesis by inhibiting tumor suppressor genes, while tumor suppressor miRNAs are downregulated in cancer and, thus, consequently, enable the overexpression of target proto-oncogenes supporting development and progression of cancer [5,11].

Previous studies have shown that miR-34a expression is related to multiple cancer types and is downregulated in a considerable number of cancers [40]. MiR-34 with its tumor-suppressing effect is able to regulate the expression of many target (proto) oncogenes implicated in tumorigenesis and cancer progression. Thus, miR-34a is traditionally considered a consistent tumor suppressor through the repression of genes that promote cell proliferation [6,41]. Under physiological conditions, miR-34a is involved in many processes to maintain cell growth and cell death and, thus, it exerts its tumor-suppressor action [42]. However, when downregulated, these tumor-suppressing processes are altered and associated with the development of cancer and other diseases and pathological states as well [11,43]. MiR-34a has frequent decreased expression in cancer due to the deletion of the locus on the chromosome 1p36 or due to epigenetic silencing (promoter CpG methylation) or p53 defective cancer cells [21,38]. Due to its downregulation in various cancers, miR-34a is considered a microRNA therapeutic candidate in cancer [6]. Notwithstanding, some studies have shown that miR-34a does not exert only a tumor suppressor role in cancer. Ma et al. in 2011 revealed the new biological oncogenic role of miR-34a upregulated in papillary thyroid cancer (PTC) tissue [41]. The authors provided an explanation of the promotion of PTC proliferation through the repression of tumor suppressor genes. They described that miR-34a regulates the expression of growth arrest specific 1 (GAS1) protein. This protein acts as a tumor suppressor. It inhibits cell growth and induces apoptosis under physiological conditions. There is emerging evidence that this mechanism of dysregulation plays an important role in the pathobiology of some cancers [41]. Recently, we described the up-regulation of miR-34a in head and neck squamous cell cancer (HNSCC) [43]. The MiR-34a–GAS1 mechanism seems to be a promising pathogenetic factor that is required to be further elucidated in HNSCC [43].

### 4.1. Head and Neck Squamous Cell Carcinoma

HNSCC encompasses malignancies of oral cavity, nasopharynx, pharynx, and larynx. It was the seventh most common cancer worldwide in 2018 with 890,000 new cases and 450,000 deaths. It represents about 6% of all cancer cases worldwide with the majority being oropharyngeal and laryngeal squamous cell carcinomas [44,45]. The etiology of HNSCC is associated with smoking, alcohol abuse, and human papillomavirus (HPV) infection [46].

The miRNAs profile represents one molecular basis of HNSCC pathogenesis that needs to be elucidated. MiR-34a expression has been studied in HNSCC from several aspects. In HNSCC, the decreased expression of miR-34a was observed previously [47]. Kozaki et al. and Scapoli et al. introduce the miRNA profile in oral cancers’ downregulation of miR-34a [48,49]. The expression levels of the miR-34 family were downregulated in HNSCC with TP53 mutations. However, differences in miR-34 family levels between groups with and without TP53 mutations were not significant since there are likely other factors activating their expression as well. Although it was documented in the cohort of 42 squamous carcinomas (HPV negative) of the oral cavity, larynx, and hypopharynx, the tumor site differences in miR-34a expression were not found [50].

However, Kalfert et al. presented significant overexpression of miR-34a in oropharyngeal carcinoma compared to adjacent healthy tissue even though many previous studies have shown low miR-34a expression in a variety of carcinomas [43,51,52]. This study introduced by Kalfert et al. revealed significant differences in miR-34a expression between oropharyngeal and laryngeal tumors as well [43]. This fact supports the hypothesis about site-specific oncogenesis in HNSCC. Moreover, the correlation between miR-34a expression and p16 status of oropharyngeal carcinoma was statistically significant [43]. P16 positivity in HNSCC is considered to be a surrogate marker of HPV etiology and the prognosis of HNSCC in p16-positive tumors is more favorable [53]. Neither an association between miR-34a upregulation and p16 positivity nor the cause of elevated miR-34a expression in HNSCC have been elucidated. P16 is known as a key regulator of cellular senescence in cooperation with senescence-associated miRNAs. MiR-34a belonging to this group could be stimulated during oncogene-induced senescence [23,43,54].

Upregulated MiR-34a may suppress HNSCC cell growth by inhibiting proteins involved in MAPK and ErbB signaling pathways. MiR-34a might be suppressed by hypoxia under the hypoxic environment of tumors in which the cancer proliferation is stimulated [55]. In addition, survivin, which is a protein with a strong anti-apoptotic effect inhibiting caspases, was explored in relationship with miR-34a in laryngeal squamous cell carcinoma. Negative regulation of survivin gene expression by miR-34a was correlated with tumor differentiation and lymphatic metastases [56]. Furthermore, the enforced expression of miR-34a in HNSCC extensively reduced cell proliferation, colony formation, and migration via target E2F3 and surviving [57]. However, the underlying mechanism of enhanced miR-34a expression remains mostly unclear. Zhang et al. documented the inhibition of human amphiregulin (AREG) by ectopic miR-34a in head and neck cancer cells [47]. AREG is a ligand for epidermal growth factor receptor EGFR and participates in the maintenance of oncogenic and metastatic properties of several solid malignancies. AREG suppression by ectopic miR-34a prevents tumor invasion in HNSCC. The MiR-34a/AREG axis might be promising molecular targets in anti-invasion/metastasis of HNSCC [47]. A comprehensive meta-analysis of miR-34a expression by Li et al. in 2018 confirmed a very important role of miR-34a in HNSCC tumorigenesis and progression as well as its potential role as biomarkers and a treatment target. Simultaneously, this study expressed the need for further research of all pathways relevant to the action of miR-34a to clarify all the mechanisms of HNSCC and their subtypes [58].

Several studies dealt with the expression of miR-34a in nasopharyngeal carcinomas. Wei et al. published the study of miR-34a in nasopharyngeal carcinoma in the relationship with clinicopathological features and prognosis. They revealed the downregulation of miR-34a that corresponded with adverse clinicopathological features (bone metastases, lymphatic metastases, TNM, and increased Ki67 proliferation) [59]. A recent study by Huang et al. presented the regulation mechanism of EMT in nasopharyngeal carcinoma. MiR-34a mediated suppression of TGF-beta/Smad4 axis to inhibit tumor progression was revealed [29]. LncRNA X inactivate-specific transcript (XIST) is found upregulated in several tumors and exerts its oncogenic function in cancers by interacting with different miRNAs. The study introduced by Song et al. in 2016 confirmed the oncogenic role of lncRNA XIST in nasopharyngeal cancer tissues and cell lines and revealed ceRNA mechanism between XIST and miR-34a as well as subsequent activation of the E2F3 signaling pathway [60]. This study confirmed the correlation between XIST expression levels and nasopharyngeal cancer prognosis, poor survival, or therapeutic outcome [60].

### 4.2. Thyroid Neoplasms

The thyroid gland tumors represent a suitable model for the study of the role of miRNAs and other biomarkers in thyroid oncogenesis because of the principal types of thyroid carcinoma (papillary and follicular) derived from the same type of cell (thyrocyte). It is clear that different pathways are involved in this neoplastic transformation. Thyroid follicular carcinoma (FTC) and thyroid papillary carcinoma (PTC) show different morphology, behavior, and clinicopathological characteristics. Moreover, less frequent medullary thyroid carcinoma (MTC) is a tumor with different histogenesis (C-cell origin) in comparison with the above-mentioned carcinomas. With respect to the diagnostic and therapeutic shortcomings associated with the thyroid tumors of follicular cell origin, new markers and molecular mechanisms are studied to improve the effective management of oncological patients. Recent studies revealed that the miRNA profile of different types of thyroid tumors seem to correspond with the pathobiology and behavior of neoplasms even though a strong overlap of miRNA profiles exist [11,61].

The most common pattern of miRNA expression in thyroid cancer cells is downregulation [62]. The frequently studied miRNAs in thyroid tumors are miR-221, miR-222, and miR-146b, which are considered markers of papillary carcinomas [63]. MiR-34a has been less commonly studied in thyroid neoplasms even though its crucial role in oncogenesis is well known. In contrast to frequent decreased miR-34a expression in a wide range of cancers [51,52], miR-34a has been reported to be upregulated in thyroid carcinomas (namely in PTC) and cell lines [64,65,66]. In the other study, the upregulation of miR-34a was confirmed in all types of differentiated thyroid carcinomas (PTC, FTC, MTC) and anaplastic thyroid carcinoma (ATC) as well as in comparison with benign tissues. Concurrently, this miR-34a expression negatively correlated with fluorodeoxyglucose (FDG) uptake [40]. Moreover, the oncogenic effect of miR-34a was described in PTC tissue and human papillary thyroid carcinoma (TPC-1) cell line via post-transcriptional repression of GAS1 [41]. The shortcoming of this study is analysis of one type of the PTC cell line only and the absence of the clinicopathological consequences of miR-34a expression in PTC tissue samples [41]. Cong et al. also revealed the relationship between miR-34a and aggressive clinical features (invasion and/or progression) of PTC as well as the significant diagnostic role of inverse combination of miR-34a and its BCL2 gene (miR-34a/BCL2) in comparison to single markers [65]. Recently, a new type of miRNA regulation, referred to as a ceRNA molecular mechanism, was introduced [25]. The lncRNA is involved in this mechanism and plays a crucial role in the interaction between them, miRNA and mRNA, and, subsequently, in tumorigenesis. Liu et al. studied this mechanism on thyroid cancer samples (not specified) and various thyroid cancers cell lines. The lncRNAXIST/miR-34a interactions through sponging miR-34a and subsequent modulation of thyroid tumor growth through MET-PI3k-AKT signaling were revealed [67]. Their findings represent a promising basis in future thyroid cancer therapy. Twelve circRNAs, 33 miRNAs, and 356 mRNAs were identified to construct the ceRNA network of PTC. These ceRNAs are critical in the pathogenesis of PTC and may also serve as future therapeutic biomarkers [67].

Medullary thyroid carcinoma is cancer derived from neuroendocrine C cells. A recent study of miRNAs in this rare neoplasm revealed high miR-34a expression having an oncogenic effect [68].

## 5. Clinical Implications of miR-34a

MiR-34a is a significant tumor suppressor that plays a considerable role in inhibiting oncogenesis and tumor progression. In recent years, many studies have shown that miR-34a has low expression in a variety of carcinomas with loss of its tumor-suppressing effect. Thus, miR-34a plays an important role in invasion, metastasis, proliferation, and EMT. That is the reason why miR-34a is considered to be a potential tumor marker and a promising cancer therapeutic candidate [35]. The contemporary limitations consist in the fact that the exact target genes of miR-34a as well as underlying pathobiological pathways of all functions in distinct tumors, remain partly unclear.

### 5.1. MiR-34a as Diagnostic and/or Prognostic Markers

MiRNA expression profiles in tumors differ from those in normal tissue and could act as a potential diagnostic, prognostic, and/or predictive markers [69,70]. However, the diagnostic value of miR-34a in cancers has been only sporadically mentioned with controversial clinical use [71,72,73].

MiRNAs upregulation and downregulation have been proven to correlate with a specific cancer phenotype playing a role as a prognostic biomarker. Prognostic value of miR-34a was studied by many researchers in various cancers, including gastric [6], colorectal [74], and breast [75]. Along with evidence for miR-34a as a prognostic and diagnostic biomarker in tissue-based samples of breast cancer, recently, serum, plasma, and urine levels of miR-34a for early diagnosis of patients with breast cancer [76,77]. However, its diagnostic/prognostic role in HNSCC and thyroid cancer remains to be more elucidated.

### 5.2. MiR-34a as Emerging Target of Therapy

The downregulation of miR-34a in various cancers is firmly established. That is the reason why the restoration of functional miR-34a can exert a therapeutic effect because of inhibition of cancer cell growth and stimulation of apoptosis and, thus, increases the drug sensitivity and/or radio-sensitivity [78]. The novel therapeutic approach via miRNA mimics enables the modulation of miR-34a expression and activity in vivo [79]. MiR-34a mimics incorporated into liposomes are currently being evaluated for cancer treatment. This is the first miRNA-associated therapeutic drug tested in a clinical trial (MRX34, NCT01829971) in April 2013 [80]. Notwithstanding, the subsequent MRX34 testing in a clinical trial in August 2016 (NCT02862145) was prematurely interrupted because of several immune-related adverse events [6]. Recently, Hong et al. tested MRX34 treatment with dexamethasone premedication in a clinical trial (NCT01829971). Although this study demonstrated a manageable toxicity profile in most patients and some clinical activity, this clinical trial was closed due to serious immune-mediated adverse events [81]. It may be difficult to predict this therapeutic effect of miR-34a, which may depend on the p53 status of the tumor and other tumor-specific genetic and epigenetic changes [19]. The success rate of the therapy depends on many factors, predominantly on the availability of clinically relevant delivery systems to avoid miRNA degradation and the potential side effects of drugs as well as some adverse immune responses [75]. Promising delivery vehicles seem to be viral vectors, lipid-based vectors, and polymeric vectors. The latter nanocarrier received attention recently because of its low immunogenicity and cytotoxicity [6].

Some cancers are difficult to completely cure by surgery alone. Radiation and conventional anti-cancer therapy are traditional modalities of cancer treatment. MiR-34a expression enhances radiation-induced apoptosis and, thus, might be possible to use as a radiosensitizer during non-small lung cell therapy. Conventional anti-cancer therapy induces miR-34a expression in human cancer cells with wild type p53, while, in p53 defect cancers, the replenishment of ectopic miR-34a may enhance the efficacy of standard cancer therapies and attenuate chemoresistance to cisplatin, 5-fluorouracil, etc. [10].

Taken together, miR-34a has been proposed for clinical application and the feasibility of this approach is currently being tested for cancer therapeutic purposes and diagnostic and prognostic potential in the future [9].

## 6. Conclusions

This review highlights the role of miR-34a in oncogenesis focusing on its tumor-suppressive functions and various regulatory mechanisms. Moreover, up-to-date findings of the involvement of miR-34a in neoplastic transformation and progression of thyroid and head and neck carcinomas are summarized. In addition, diagnostic, prognostic, and therapeutic promises and expectations of miR-34a are introduced. Further understanding and revealing the molecular mechanism underlying thyroid and head and neck cancer are indispensable for the development of effective diagnosis and treatments. Although the mechanism of miR-34a in tumors has been initially explored, further clarification of its role in tumorigenesis needs to be elucidated. Therefore, novel basics for targeted therapy are examined. It is noted that miR-34a usually targets different genes in different tumor types, which suggests miR-34a may be involved in the distinct signaling pathways in specific tumors. Notably, it is necessary to take into consideration that the involvement of miR-34a in oncogenesis is site-specific and type-specific requiring studying various cancers separately.
